# EEG/EOG/EMG data from a cross sectional study on psychophysiological insomnia and normal sleep subjects

**DOI:** 10.1016/j.dib.2017.09.033

**Published:** 2017-09-21

**Authors:** Mohammad Rezaei, Hiwa Mohammadi, Habibolah Khazaie

**Affiliations:** Sleep Disorders Research Center, Kermanshah University of Medical Sciences, Kermanshah, Iran

**Keywords:** Sleep dataset, Psychophysiological, Insomnia, EEG

## Abstract

The data presented here had been originally collected for a research project entitled ‘Sleep EEG spectral analysis in psychophysiological insomnia and normal sleep subjects’. This article describes the data of 11 subjects, referred to Sleep Disorders Research Center (SDRC) in Kermanshah, Iran. The data includes 14 EEG, 6 EOG, and 3 EMG channels, with a sampling ratio of 256 Hz. It includes power spectral features in segments of 30 s for each channel, and nonlinear analysis parameter. Also, the complete demographic and polysomnography specifications are attached.

**Specifications Table**

**Table:** 

Subject area	*Neuroscience, Neurobiology*
More specific subject area	*Psychiatry, sleep, psychophysiological insomnia*
Type of data	*Table, text file, m-file, mat file,*
How data was acquired	*Polysomnography, Matlab software*
Data format	*Filtered, analyzed,*
Experimental factors	*Sleep questionnaire were used for subjective features. Age, gender, height, weight, education, marriage, and body mass index were used as covariates.*
Experimental features	*Power spectrum includes delta, theta, alpha and beta bands. Parameters from nonlinear analysis (Poincare's map and standard descriptors).*
Data source location	*Samples were collected in the Sleep Disorders Research Center in Kermanshah University of Medical Science.*
Data accessibility	*The dataset is freely available at**[1]**for any academic, educational, and research purposes.*
	http://dx.doi.org/10.17632/3hx58k232n.4

**Value of the data**

• The raw data could be processed using algorithms and other procedures during future researches.
• The data represents 8 h of sleep signals (EEG, EOG, and EMG) from 22 subjects; including 11 psychophysiological insomniacs and 11 normal subjects.
• Psychophysiological insomnia is a more prevalent sleep disorder, which leads to clinically significant impairment in social, occupational, and cognitive functions.
• The data can also be used to assess the EEG Sleep Pattern in psychophysiological insomnia patients as well as good sleepers.
• The diagnosis was performed by a sleep clinician, based on subjective and objective sleep features.

## 1 Experimental design, materials and methods

### 1.1 Participant

A total of 22 subjects that includes 8 males (36.36%) and 14 females (63.64%) aged between 18 and 63 years (43.2±14.2) were recruited for participation. Out of the participators, 11 patients were suffering from psychophysiological insomnia (18.18% male; age: 44±13.2 years; Body Mass Index (BMI): 26.6±3.7 kg m^−2^) and 11 subjects had normal sleep pattern (54.5% male; age: 42.4±15.4 years; BMI: 27.53±4.24 kg m^−2^).

The patients were selected from people referred to SDRC, due to insomnia complaints. Normal sleep subjects were recruited from the general population. A detailed written consent was obtained from all participants. Both patients and normal subjects completed their demographic and medical history checklists, including substance and alcohol check as well as psychiatric disorders. For selection of normal subjects, candidates had to first complete the Pittsburgh questionnaire. Preliminary selection was done based on these results. Then, they were further tested using Polysomnography. Finally, subjects who cleared the PSG test round, were selected.

### 1.2 Procedure

All subjects underwent a one-night polysomnography (PSG) test with the help of SOMNOscreen device called SOMNOscreen™ plus PSG produced by SOMNOmedics GmbH, Germany. The duration of the test was 8 h (23:00–07:00 h), as per standard protocol at SDRC of KUMS, Iran. A day before appointment, the subjects were invited to sleep in the laboratory of SDRC. They were advised against consuming any tea, coffee, heavy diet or cigarette. Sleeping during the day was also prohibited. Upon arriving at the laboratory, the height and weight of the subject was measured by an experienced personnel. Next, the subjects and participants completed the Pittsburgh questionnaire followed by a detailed briefing on PSG procedures. The measurement of PSG was based on the American Academy of Sleep Medicine guidelines. The polysomnography room was cleaned from artefacts like auditory and visual noises, based on standards [1].

**Fig. 1:**
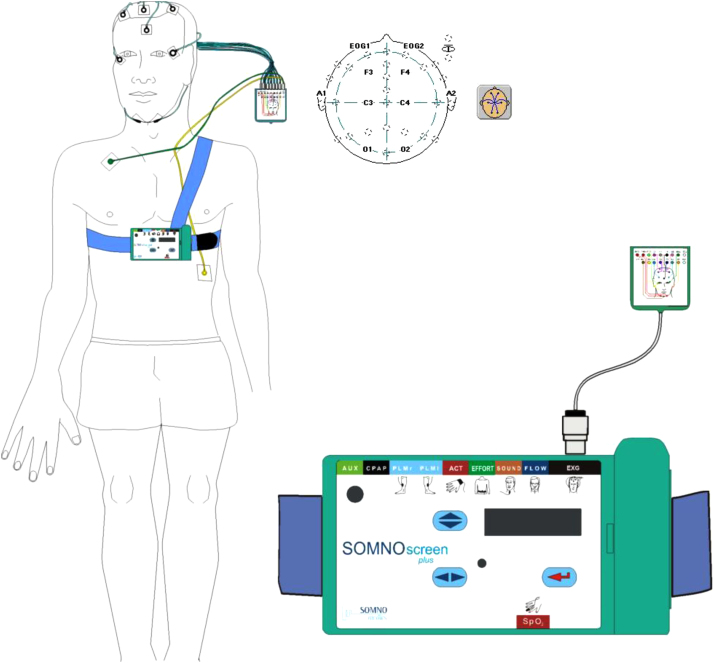
Electrode montage corresponding to the international 10–20: Sites included frontal (F3, F4), central (C3, C4), and occipital (01, 02) placements of the International 10–20 System.

**Fig. 2:**
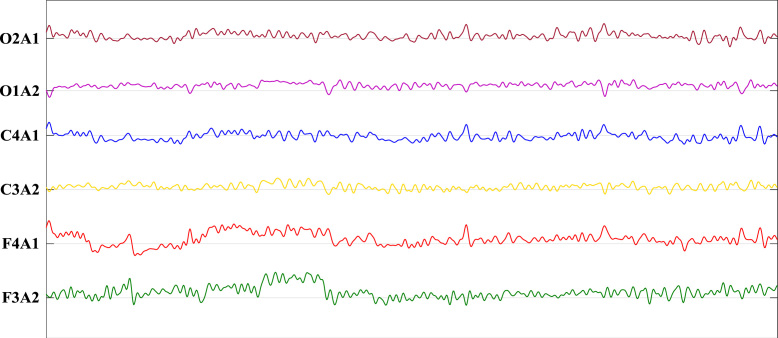
6 EEG channels sampled at 256 Hz using SOMNOscreen PSG (SOMNOscreen TM plus PSG+, SOMNOmedics GmbH, Germany). This shows the voltage in microvolts on each channel referenced to the left or right earlobe (A1, A2) over a 4 s window.

24 recording electrodes were prepared, including 14 electroencephalogram channels (C4A1, C3A2, F3, F4, C3, C4, A1, A2, O1, O2, F3A2, F4A1, O1A2, O2A1), 6 electrooculogram channels (EOG1, EOG2, EOG1A1, EOG2A1, EOG1A2, EOG2A2), 3 electromyogram channels (EMG, EMG1, EMG2), and ECG channel. All the recordings were sampled at the rate of 256 Hz. EEG was recorded using Ag/AgCl electrodes, as per the International 10–20 System of Electrode Placement, as shown in Fig. 1. Recorded EEG signals is also shown in Fig. 2.

Both psychophysiological insomnia and normal sleep were determined after clinical interview and careful study of the data obtained from PSG test by an experienced psychiatrist, trained in sleep medicine and PSG. Subjective information was obtained from clinical interview using Pittsburgh Sleep Quality Index (PQSL) [2], [3].

All raw datasets were stored in the European Data Format (EDF) format, one file per subject. For example, the file ‘Normal_Subject_xx’, contains the raw data from normal subject number ‘xx’. Similarly, for a patient suffering from psychophysiological insomnia, the file would be ‘Raw_Signal_Psychophysiological_ Insomnia_xx’. Description of all participants were recorded in ‘PSG_Psycho_Normal.xlsx’. The outputs from polysomnography were collected at ‘PSG_Outputs.rar’. Data file names and their descriptions are listed in Table 1.

**Table 1:** Data file names and their descriptions.

Name	Description
Arousal.txt	Time of arousal events
REM.txt	Time of REM events
Sleep Profile Reliability.txt	Reliability of the sleep profile
Sleep Profile.txt	Time of events including Stage 4, Stage 3, Stage 2, Stage 1,Rem,Wake,Movement.
Spindle K.txt	Time of events sleep spindle and K_Complex.

Two files, namely PSD_Normal_Subjects and PSD_Psycophysiological_Insomnia, contain the power spectral density analyses for all subjects. In these files, the data is arranged in the form of cell arrays, where each row represents a channel and each column represents a 30 s epoch. Also, each cell contains a two-column matrix; the first column represents frequency and the second, power. The power spectrum analysis is commonly used in the study of biological signals, to calculate the frequency power [4]. Power spectrum analysis was conducted using Fast Fourier transform (FFT) in the range of 0.1–35 Hz, continually [5]. This transform is defined as follows:

(1) $$Y\left(k\right)=\sum_{j=1}^{n}X(j)\times W_{n}^{(j-1)(k-1)}$$

In this data collection project, frequency resolution in power spectrum analysis was 0.5 Hz, which is defined as Fs/N in FFT [6]. As polysomnography analysis is separated by 30 s time intervals, the band's power was also extracted from these 30 s epochs.

The EEG data was filtered using the band pass method between 0.1 Hz and 35 Hz. It should be noted that signal filtering was done by SOMNOscreen™ plus instrument and the filtered data was exported only, and not completely raw data. In the next step, large artefacts due to electromyography activity, horizontal eye movement or ECG artefact were removed using independent component analysis (ICA). This method was considered in the past decade. Independent signal extraction from mixed signals is one of its applications. The ICA literature is divided into two major categories: practical algorithms and theoretical analyses. ICA could separate activities stemming from the most favourable parts of the brain by using their independent components [7].

With further spectral features, the present data has some important benefits to sleep clinicians and researchers, who are unaware of spectral analysis. They would have considerable findings by download and statistical analysis of spectral features.

### 1.3 Poincare map

The Poincare's map, as a nonlinear analysis, is valuable, as it could reveal the nonlinear aspects of the data collection [8]. Therefore, the challenge lies in recording temporal information of the plot quantitatively. The standard descriptors used for quantifying the Poincare's map, measure the impure variability of the time series data. Standard deviations across the line of identification (*SD*_2_) and perpendicular to the line of identification (*SD*_1_), represent the magnitude of the major and minor axes of the ellipse, respectively. *SD*_1_ represents the *SD* of the instantaneous short term variability. *SD*_2_ represents the *SD* of long-term variability. *SD*_1_ and *SD*_2_ support the potent data and also, the proportion of *SD*_1_ to *SD*_2_ has been suggested as strongly indicator [9]. Descriptors *SD*_1_ and *SD*_2_ can be defined as:

(2) $$SD_{1}=\frac{\sqrt{2}}{2}\times SD(x_{n}-x_{n+1})$$

(3) $$SD_{2}=\sqrt{2\times SD{\left(x_{n}\right)}^{2}-\frac{1}{2}\times SD{\left(x_{n}-x_{n+1}\right)}^{2}}$$

where *SD* is a standard deviation of the time series.

To plot the Poincare's map — summation, maximum, and standard deviation of the band's power in each epoch was calculated and saved, as time series data. Then, the standard descriptors were calculated and the Poincare plots were extracted from the statics result of the band's power. Each Poincare plot was constructed with the *X*-axis illustrating the statics result, at a specific epoch (*x*(*k*)), and the *Y*-axis illustrating the statics result after a specified epoch delay (*x*(*k*+1)).

**Fig. 3:**
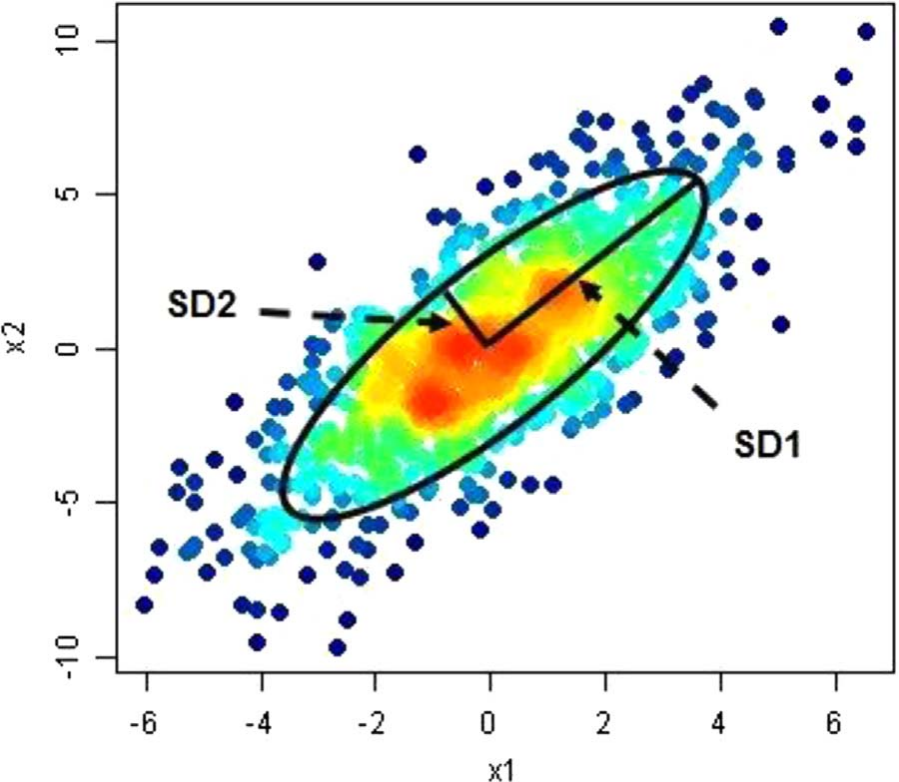
A typical Poincare plot. The horizontal axes demonstrate the illustrating the statics result at a specific epoch (*x*(*k*)) and the vertical axis illustrating the statics result after a specified epoch delay (*x*(*k*+1)). An ellipse is fitted to the data points and the Poincare plot descriptors are measured by estimating the *SD* perpendicular to the diametric line was defined as *SD*_1_, and the *SD* along the diametric line was defined as *SD*_2_ and ratio of the *SD*_1_/*SD*_2_ of the fitted ellipse.

We calculated the *SD* of statics result dispersion along with the diametric line and the *SD* of the power dissipation perpendicular to the diametric line, to present more quantitative data on the repartition of statics result in the Poincare plots (Fig. 3). The *SD* perpendicular to the diametric line was defined as *SD*_1_ and the *SD* along the diametric line was defined as *SD*_2_. *SD*_1_ is the level of instantaneous variability and demonstrates the variability from one epoch to the next. In contrast, *SD*_2_ demonstrates the power variability across all the epochs. We then defined the *SD*_1_/*SD*_2_ ratio that is a potent candidate, reflecting the psychophysiological disorder depth, which has been used to specify the clarity and linearity of the scatter pattern.
